# A review of the family Trichopolydesmidae in North Africa with a description of a new species from Tunisia

**DOI:** 10.3897/zookeys.786.28270

**Published:** 2018-09-26

**Authors:** Nesrine Akkari, Jean-Paul Mauriès

**Affiliations:** 1 Dritte Zoologische Abteilung, Naturhistorisches Museum Wien, Burgring 7, A-1010 Wien, Austria Naturhistorisches Museum Wien Wien Austria; 2 Muséum National d’Histoire Naturelle, Dpt. Origines & Evolution, Section Arthropodes, 61 rue Buffon F- 75005 Paris, France Muséum National d’Histoire Naturelle Paris France

**Keywords:** *
Haplocookia
*, *
Heterocookia
*, identification key, North Africa, new species, taxonomy, Tunisia, updated checklist

## Abstract

A new species of the genus *Haplocookia* Brölemann, 1915 is described from Cap Bon Peninsula in Tunisia (North Africa) and a historical account of the poorly understood genera *Haplocookia* and *Heterocookia* Silvestri, 1898 is provided. Comments on the taxonomy of the family Trichopolydesmidae are presented, as well as an identification key to the trichopolydesmid species hitherto known from North Africa and an updated list of the Polydesmida in the region.

## Introduction

The order Polydesmida Leach, 1815 is represented in North Africa with five families, nine genera, and 22 species (see list below). Most of these species are endemic, marginally studied, and the taxonomy of several species and genera remain far from adequate (see Brölemann 1921, Schubart 1960, Tabacaru 1975, Mauriès 1984, Hoffman 1980, Akkari and Enghoff 2011, Enghoff et al. 2015). Among these, the genera *Haplocookia* Brölemann, 1915 and *Heterocookia* Silvestri, 1898, represented with four endemic species, have particularly been subject to taxonomic controversies and remain poorly understood in comparison with the rest of the polydesmidans in this region.

The genus *Haplocookia* is characterised by a deeply divided gonopod telopodite bearing short and simple processes. It was first established by Brölemann (1915) to accommodate *Haplocookiamauritanica*, he then described from Kabylie, Algeria. The genus remained monotypic until Schubart (1960) described *Haplocookiafranzi* from Morocco. *Haplocookiafranzi* Schubart, 1960 differs from the type species in the shape and processes of the distal part of the telopodite.

The genus *Heterocookia* was described much earlier, based on a species collected and described from Aϊn Draham Region in northwestern Tunisia by Silvestri (1896), *Heterocookianovator* (Silvestri, 1896). The genus counts a second species, *Heterocookiatunisiaca* Ceuca, 1967 described from Le Kef (Ceuca 1967). Both genera were first placed with six other genera in the tribe Trichopolydesmini (Brölemann 1915). Subsequently, Attems (1940) considered *Haplocookia* as a junior synonym of *Heterocookia* Silvestri, 1898, listing the species *mauritanica* under the genus *Heterocookia* in his Tierreich volume on Polydesmoidea. Two decades later, Schubart (1960) re-established *Haplocookia* as a valid genus, placed it in the family Vanhoeffeniidae Attems, 1914 and described a third species, *H.franzi* from several localities in Morocco. After two more decades, Hoffman (1980) also considered *Haplocookia* as a valid genus although he listed only one of the two described species, and assigned both *Haplocookia* and *Heterocookia* to the family Polydesmidae Leach, 1815. Mauriès (1984) was the last to discuss the taxonomy of the genus *Haplocookia* and he recommended placing it back in the family Trichopolydesmidae as previously suggested by Brölemann (1915) and Tabacaru (1975). Golovatch (2013) accepts both *Haplocookia* and *Heterocookia* in Trichopolydesmidae but in the latest taxonomic overview of the order Polydesmida, the genus *Haplocookia* is absent, whereas *Heterocookia* is listed under the family Trichopolydesmidae (Enghoff et al. 2015).

In this paper, we shed light on this obscure genus, describe a new species from Tunisia, *Haplocookiaenghoffi* sp. n., and we further provide an updated checklist of the polydesmidan fauna of North Africa and an identification key to the species of *Haplocookia* and *Heterocookia* in this region.

## Material and methods

The material of the new species was collected by NA, stored in 70% ethanol, and deposited in the Muséum national d’Histoire naturelle (**MNHN**), Natural History Museum of Denmark, Zoological Museum – University of Copenhagen (**ZMUC**), and Naturhistorisches Museum Wien (**NHMW**). Type material of *Haplocookiamauritanica* (**MNHN**) was examined for comparison. General characters were studied with a Wild Heerbrug 308700 stereomicroscope from Zeiss. Measurements and drawings were obtained using a camera lucida of a compound microscope Axioskop from Zeiss. Parts of some specimens were mounted on microscope preparations in lactic acid for examination. Micrographs were made in NHMW with a Nikon DS-F2.5 camera mounted on a Nikon SMZ25 stereomicroscope, using NIS-Elements Microscope Imaging Software with an Extended Depth of Focus (EDF) patch. All images were processed with Adobe Photoshop CS6 and assembled in Adobe InDesign CS6.

## Taxonomy

### Order Polydesmida Leach, 1815

#### Family Trichopolydesmidae Verhoeff, 1910

##### Genus *Haplocookia* Brölemann, 1915

###### Haplocookia enghoffi sp. n.

Taxon classificationAnimaliaPolydesmidaTrichopolydesmidae

http://zoobank.org/C2E5B414-CA4D-4710-9515-93851F54D9DD

Figs 1 , 2 , 3

####### Material.

**Holotype**. Male, Tunisia, Cap Bon peninsula, Nabeul district, Jebel Abderrahman, Tunisia, 28.11.2004, N. Akkari leg. (MNHN – JC 380). **Paratypes**. 2 males, same data as holotype, N. Akkari leg. (MNHN – JC 380); 2 males, same data as holotype, N. Akkari leg. (NHMW 9366; NHMW 9367); 1 male, same data as holotype, N. Akkari leg. (ZMUC 00039891).

####### Additional material studied.

*Heterocookianovator*, 1 male, Tunisia, Gov. Béja, Jebel El Jouza Amdoun, coll. & det. N. Akkari, MNHN; *Heterocookiatunisiaca*, 1 male, Algeria, wilaya El Tarf, El Kala, coll. Kahina Houd-Chaker, det. J.-J. Geoffroy, MNHN.

####### Diagnosis.

A small polydesmidan of the genus *Haplocookia*, differing from its congeners in the shape of the distal part of the gonopod telopodite having simple curved processes.

####### Etymology.

The species epithet honours Prof. Henrik Enghoff, a leading expert in myriapod systematics, author of major works on millipede taxonomy, and always a dear friend.

####### Description.

**Figure 1.**
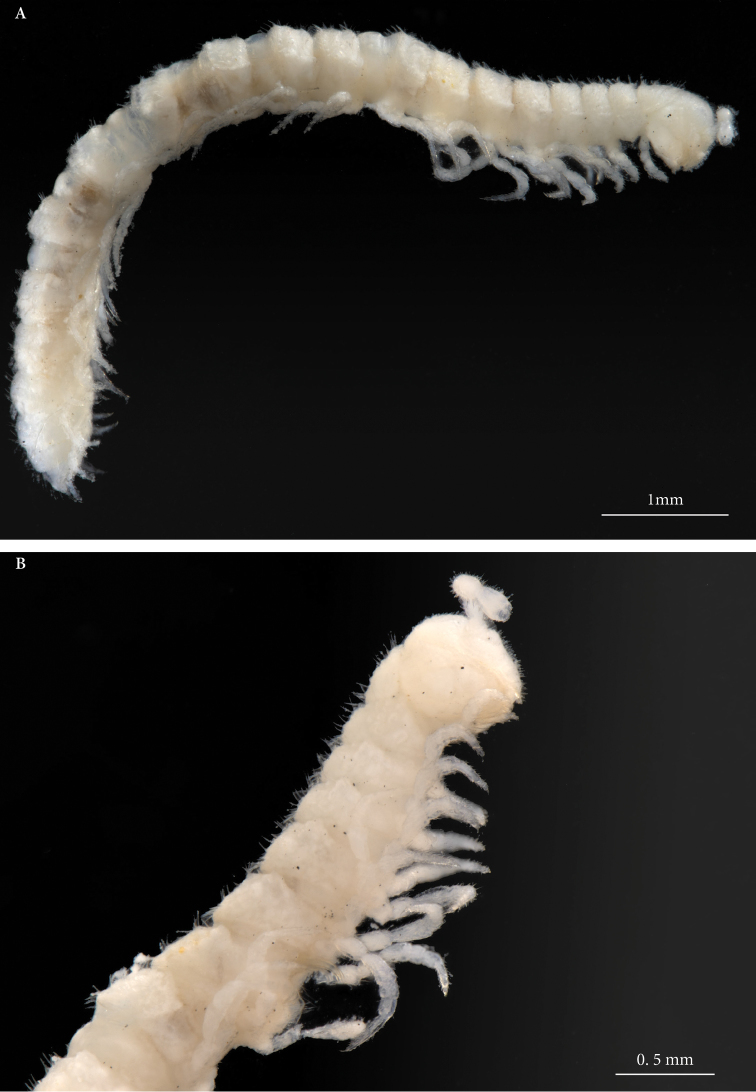
*Haplocookiaenghoffi* sp. n. ♂ paratype NHMW 9366: **A** Habitus, lateral view **B** Head and first body rings, lateral view.

(all measurements in mm). Pale, almost white (Figure 1), 20 body rings; length: 8−8.6 mm, width of the 10^th^ metazonite, including paranota: 0.83−1.18; prozonite: 0.62−0.69.

**Figure 2.**
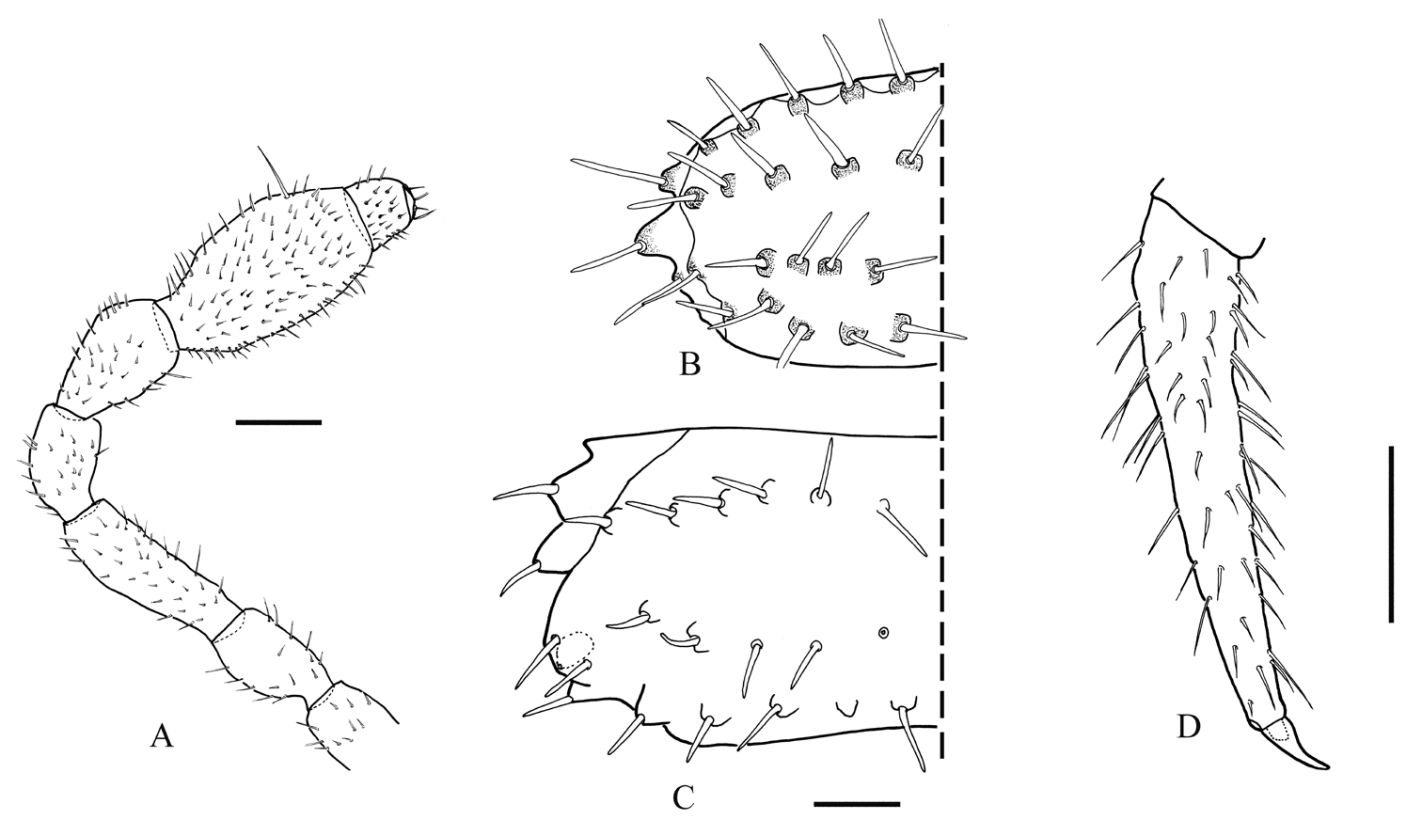
*Haplocookiaenghoffi* sp.n. ♂ paratype MNHN- JC 380: **A** Antenna **B** Collum **C** 10^th^ metatergite bearing ozopore **D** leg, tarsus and apical claw. Scale bar 0.1 mm.

***Head*** occipital furrow not clear; mandibles and gnathochilarium with many small and regularly distributed setae, labrum with three teeth. Antenna (Figure 2A) 1.21 mm long, articles: 1^st^: 0.12, 2^nd^: 0.15, 3^ed^: 0.24, 4^th^: 0.16, 5^th^: 0.18, 6^th^: 0.26, 7^th^& 8^th^: 0.10, no special characters observed on 6^th^antennomere (only the usual external long seta).

***Collum*** (Figure 2B) semicircular, not broader than head, flattened, with four irregular transverse rows of tubercles bearing stout and long setae, paranotal edges incised into three well-developed lobes, each one bearing 1 seta.

***Metaterga*** (Figure 2C) with three transverse rows of 10−14 tubercles each bearing a short and stout seta (anterior and posterior rows with ten tubercles each, median row with variable number), median row closer to posterior one.

***Paranota*** (Figure 2C) well expanded dorsolaterally, with four incised lobes bearing one long and stout seta each. Ozopore large, round and lying between the two posterior metatergal rows, present on rings 5, 7, 9, 10, 12, 13, 15 −19.

***Legs*** (Figure 2D) without special features, articles: coxa: 0.17, prefemur: 0.20, femur: 0.08, postfemur: 0.10, tibia: 0.15, tarsus: 0.10, apical claw circa three times longer than broad (length: 0.04, basal width: 0.01).

***Telson*** with two transverse rows of tubercles bearing long and strong setae, epiproct almost triangular, with relatively long setae.

**Figure 3.**
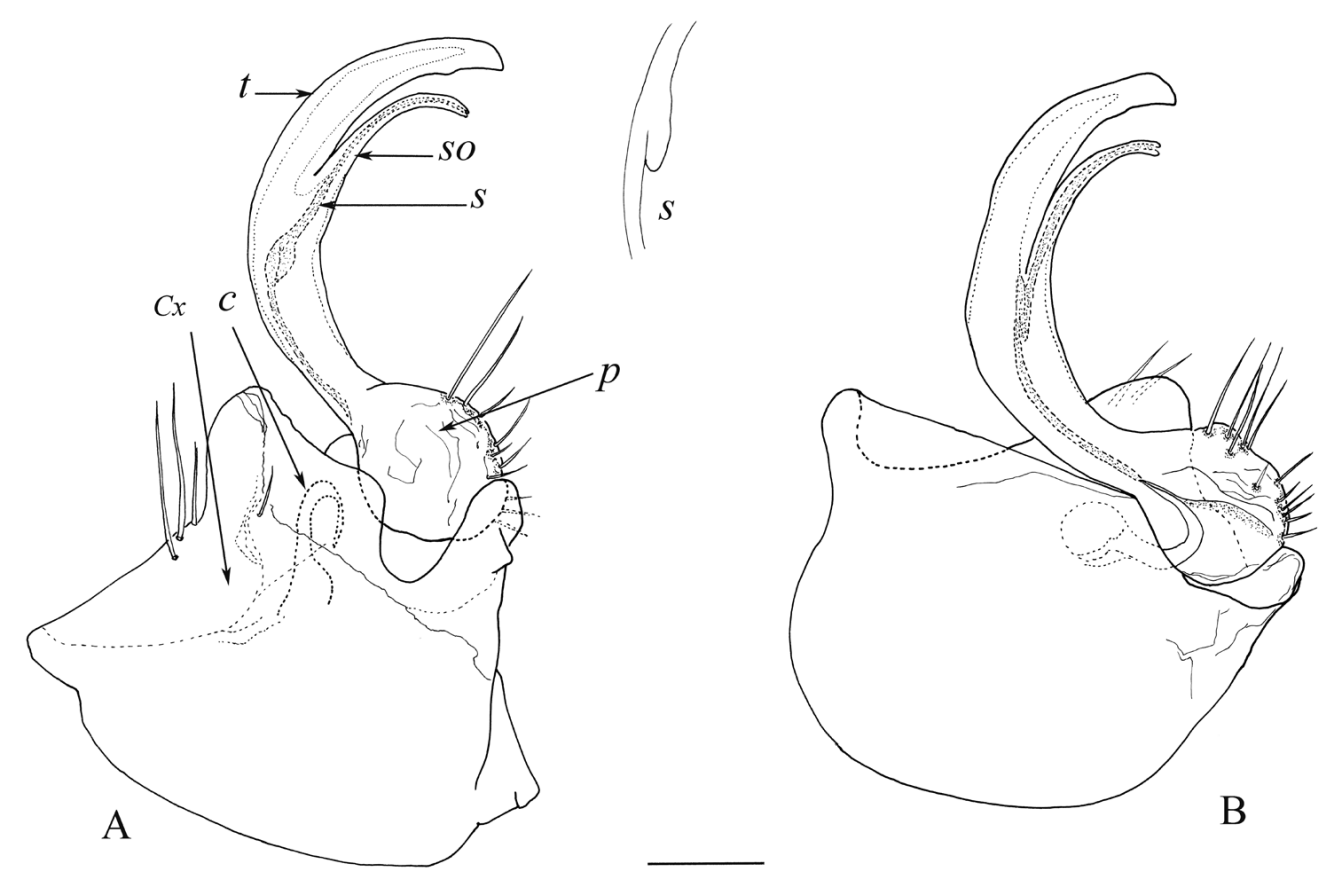
*Haplocookiaenghoffi* sp. n. ♂ paratype: **A** Left gonopod, mesal view **B** Left gonopod, postero-lateral view. Abbreviations: C cannula, Cx Coxa, p prefemur, S seminal groove, So solenomere, t tibiotarsus. Scale bar 0.1 mm.

***Gonopods*** (Figure 3). Coxa (*Cx*) well-developed, hemispherical, internal margin not indented, external border extended in a large anterior rounded lobe with 2 long and 1 shorter setae seen in posterior view. Prefemoral part (*p*) with strong setae, medially folded and sheltering basal opening of seminal groove. Cannula (*C*) concealed in coxa, its tip entering mesal fold of the prefemur, where seminal groove (*S*) arises. Distal part of telopodite divided into solenomere (*So*) and tibiotarsus (*t*). Tibiotarsus simple, relatively broad and apically bent, with barely perceptible blunt bump on internal margin. Solenomere (*So*) slender and bent bearing the opening of the seminal groove at apex. Seminal groove (*S*) uniformly broad from femoral basis up to apex of solenomere, noticeably thickening at femoral level, just above bifurcation of telopodite.

####### Comments.

*H.tunisiaca* is reported here for the first time from Algeria.

## Discussion

### Notes on the North African trichopolydesmids

Except for the special structure of the seminal groove (a small bulb-like extension, reminding of genus *Polydesmus*), the gonopod of *Haplocookiaenghoffi* sp. n. is built in the same way as that of *H.mauritanica* and *H.franzi*, with a typically polydesmoid crescent-shaped telopodite arising from a large coxa (Figs 3, 5A, B). In all three species, the telopodite is divided into a tibiotarsus and a slender solenomere. However, these two processes show different configurations in the three species (Figs 3, 5A, B). In *H.mauritanica*, the two processes separate at the apical third of the telopodite and the solenomere is a very slender process orthogonal to the main telopodite axis. In *H.franzi*, the solenomere is a small and elongated branch, slightly bent and forked, laterally protected by a larger tibiotarsus. The telopodite is clearly indented in *H.franzi*, presenting a subapical triangular tooth in *H.mauritanica*, and only a small subapical blunt bump in *H.enghoffi* sp. n.

**Figure 4.**
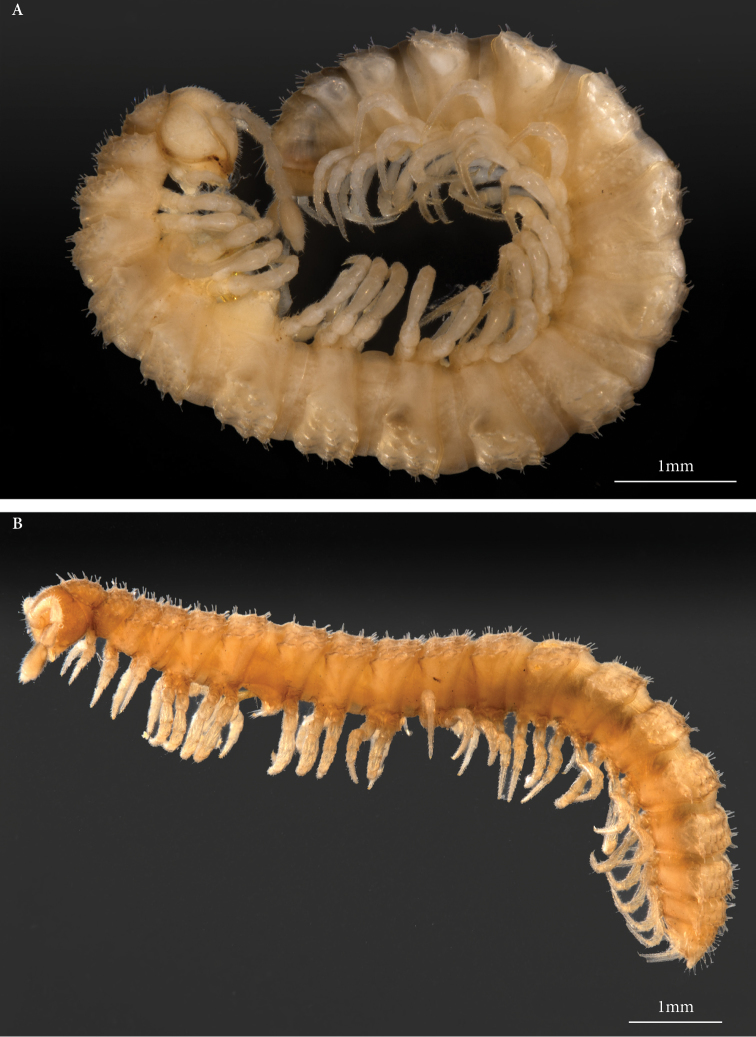
*Heterocookianovator* (Silvestri, 1896) and *Heterocookiatunisiaca* Ceuca, 1967, habitus. **A***Heterocookianovator* (Tunisia, Gov. Béja, Jebel El Jouza Amdoun, coll. & det. N. Akkari, MNHN) **B***Heterocookiatunisiaca* (Algeria, wilaya El Tarf, El Kala, coll. Kahina Houd-Chaker, det. J.-J. Geoffroy, MNHN).

**Figure 5.**
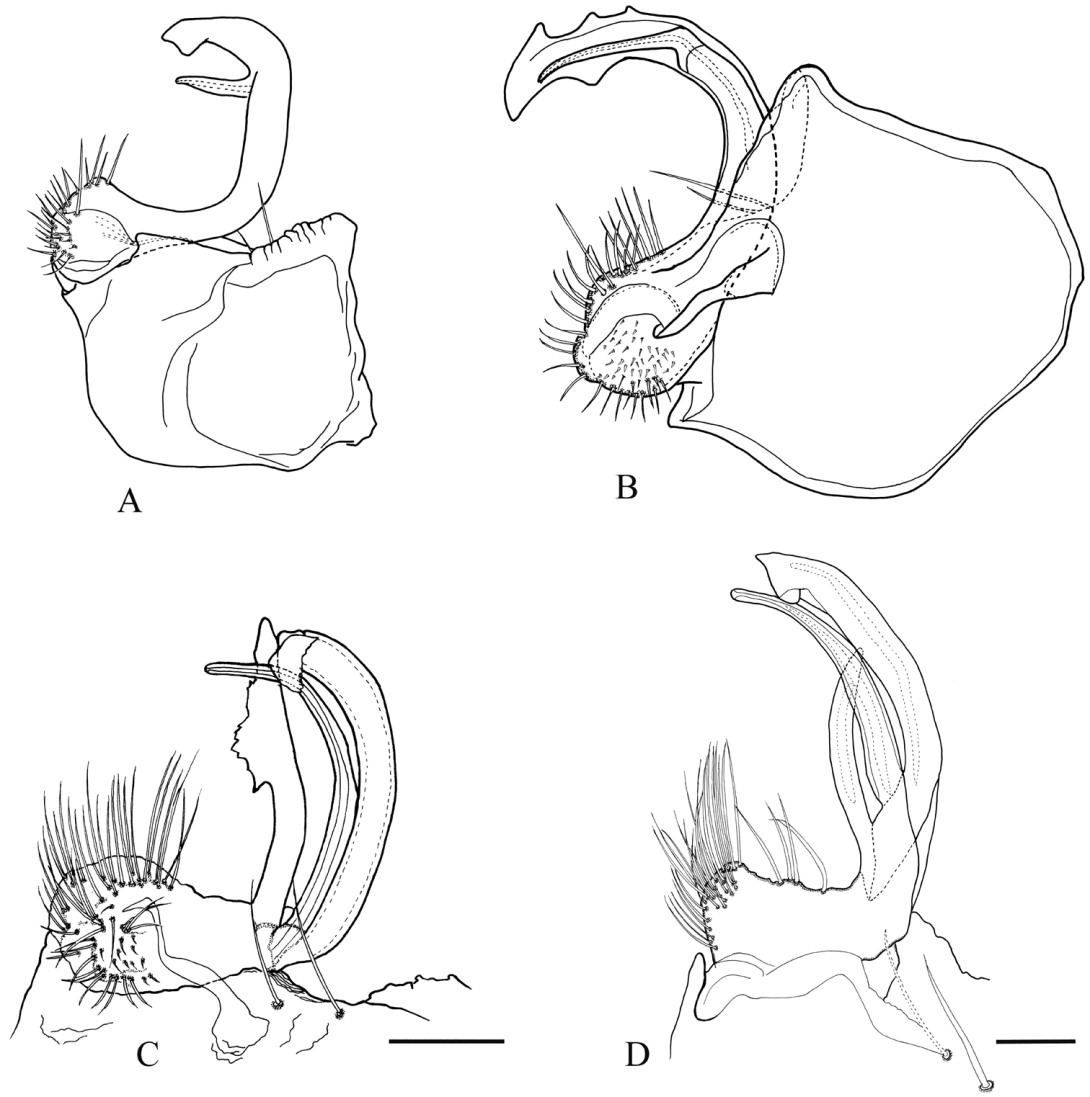
North African Trichopolydesmidae, right gonopod in postero-lateral view: **A***Haplocookiamauritanica* Brölemann, 1915 (redrawn after Brölemann 1915) **B***Haplocookiafranzi* Schubart, 1960 (redrawn after Schubart 1960) **C***Heterocookianovator* (Silvestri, 1896) **D***Heterocookiatunisiaca* Ceuca, 1967. Scale bar 0.1 mm.

The genus *Heterocookia* includes two species from Tunisia, viz. *H.novator* (Figure 4A) and *H.tunisiaca* (Figure 4B), the latter is recorded here for the first time from El Tarf in Algeria. Both species are larger than the *Haplocookia* species despite sharing the same external characters. Their gonopods (Figure 5C, D) are characterised by a deep ramification of the telopodite, which clearly shows three slender processes composed of a simple solenomere, a more complex tibiotarsus, and a third process.

### Notes on the family Trichopolydesmidae

The taxonomy of the family Trichopolydesmidae has remained controversial. Verhoeff (1910) erected the subfamily Trichopolydesminae for the two genera *Trichopolydesmus* Verhoeff, 1898 and *Bacillidesmus* Attems, 1898. Later, Attems (1926, 1940) placed the genus *Trichopolydesmus* in the family Vanhoeffeniidae Attems, 1914. Although Verhoeff (1943) reestablished the Trichopolydesmidae as a full family, Schubart (1960) described *Haplocookiafranzi* in the family Vanhoeffeniidae. The families Vanhoeffeniidae and Sphaerotrichopodidae were synonymised with Dalodesmidae by Jeekel (1956). Tabacaru (1975, 1980) provided a new circumscription of Trichopolydesmidae based on the works of Verhoeff (1943), Ceuca (1958, 1974) and Kraus (1957). In his survey, Tabacaru (1980) gathered in the same group five genera: the type genus *Trichopolydesmus* [including the subgenus Banatodesmus Tabacaru, 1980 he then described, and which was later treated by Mauriès (1983, 1984) as a full genus and placed in the Fuhrmannodesmidae Brölemann, 1916] together with *Bacillidesmus*, *Galliocookia* Ribaut, 1955, *Verhoeffodesmus* Strasser, 1959 and *Napocodesmus* Ceuca, 1974. Following the same logic, Mauriès (1980, 1984) established a list and an identification key for the nine genera in the Trichopolydesmidae, adding *Haplocookia*, *Cottodesmus* Verhoeff, 1936, *Occitanocookia* Mauriès, 1981 [1980 in Nomenclator 3] and *Ingurtidorgius* Srasser, 1974 to the five above mentioned genera.

Almost simultaneously, Hoffman (1980) placed the genera *Galliocookia*, *Haplocookia* and *Heterocookia* in the family Polydesmidae and separated a small number of genera from the Fuhrmannodesmidae, placing them within three subfamilies of Trichopolydesmidae, viz. Trichopolydesminae (*Trichopolydesmus*), Bacillidesminae Verhoeff, 1910 (*Bacillidesmus* and *Napocodesmus*) and Ingurtidorgiinae Strasser, 1974 (*Ingurtidorgius* Strasser, 1974). Mauriès (1984) included four more genera in the Trichopolydesmidae, viz. *Haplocookia*, *Verhoeffodesmus*, *Cottodesmus* and *Occitanocookia*.

Ten years later, Simonsen (1990) underlined a clear geographical discontinuity between the Euro-Mediterranean and the Afrotropical taxa, placing them in Trichopolydesmidae and the Fuhrmannodesmidae, respectively, which was supported subsequently by Shelley (2003). Recently, Shear (2011) provided a list of Trichopolydesmoidea, where the family Trichopolydesmidae was not mentioned, very likely merged with the Fuhrmannodesmidae. Among the latest contributions, Golovatch (2013) reclassified the superfamily Trichopolydesmoidea, presented a diagnosis for the family Trichopolydesmidae, based on male sexual characters and provided a new circumscription of the family in which he included the Fuhrmannodesmidae, Macrosternodesmidae Brölemann, 1916, Mastigonodesmidae Attems, 1914 and Nearctodesmidae Chamberlin and Hoffman, 1950. This same classification was also adopted by Enghoff et al. (2015) in their classification of the Polydesmida. The Trichopolydesmidae currently includes around 75 genera and 140 species (see Golovatch 2013), among which nearly 20 genera and 40 species with Euro-Mediterranean distribution, and two genera and five species in North Africa.

### Key to North African species of Trichopolydesmidae based mostly on male gonopods

**Table:** 

1	Pale species (Fig. 1). Gonopod with two processes (Figs 3, 5A, B)	***Haplocookia* 2**
–	Pigmented species (Fig. 4). Gonopod with three processes (Figs 3, 5C, D)	***Heterocookia* 4**
2	Tibiotarsus with strong subapical or apical indentations (Figs 3, 5A, B)	3
–	Tibiotarsus with a faint subapical projection (Fig. 3)	***Haplocookiaenghoffi* sp. n.**
3	Tibiotarsus with apical indentation (Fig. 5A), solenomere short and bent orthogonally to tibiotarsus	*** Haplocookia mauritanica ***
–	Tibiotarsus with subapical marginal indentations; solenomere slender and bent in same plane as tibiotarsus (Fig. 5B)	*** Haplocookia franzi ***
4	Tibiotarsus with an upturned tip. Accessory process with lateral serrated expansion (Fig. 5C)	*** Heterocookia novator ***
–	Tibiotarsus with cleaver-shaped tip. Accessory process slender, without serrations (Fig. 5D)	*** Heterocookia tunisiaca ***

## List of species of Polydesmida in North Africa

Family Polydesmidae Leach, 1815

*Archipolydesmuschreensis* Abrous-Kherbouche & Mauriès, 1996

*Archipolydesmusfodili* Abrous-Kherbouche & Mauriès, 1996

*Archipolydesmuskabylianus* Abrous-Kherbouche & Mauriès, 1996

*Archipolydesmusmaroccanus* Attems, 1898

*Polydesmusdismilus* (Berlese, 1891)

*Polydesmusproximus* (Latzel, 1884)

*Polydesmussuperus* (Latzel, 1884)

Family Pyrgodesmidae Silvestri, 1896

*Rharodesmuscherifiensis* Schubart, 1960

*Rharodesmustabarkensis* Akkari & Enghoff, 2012

?*Tonodesmusbolivari* Silvestri, 1923

Family Trichopolydesmidae Verhoeff, 1910

*Haplocookiaenghoffi* sp. n.

*Haplocookiafranzi* Schubart, 1960

*Haplocookiamauritanica* Brölemann, 1915

*Heterocookianovator* (Silvestri, 1896)

*Heterocookiatunisiaca* Ceuca, 1967

Family Xystodesmidae Cook, 1895

?*Melapheblainvillei* (Eydoux & Gervais, 1836)

*Melaphemauritanica* (Lucas, 1844)

*Macellolophusrubromarginatus* (Lucas, 1846)

Family Paradoxosomatidae Daday, 1889

*Boreviulisomaliouvillei* Brölemann, 1928

*Eviulisomaabadi* Mauriès, 1985

*Oranomorphaguerinii* (Gervais, 1836)

*Stosateaitalica* (Latzel, 1886)

## Supplementary Material

XML Treatment for Haplocookia enghoffi
